# The advances in adjuvant therapy for tuberculosis with immunoregulatory compounds

**DOI:** 10.3389/fmicb.2024.1380848

**Published:** 2024-06-20

**Authors:** Jie Mi, Xueqiong Wu, Jianqin Liang

**Affiliations:** ^1^Beijing Key Laboratory of New Techniques of Tuberculosis Diagnosis and Treatment, Institute of Tuberculosis Research, Senior Department of Tuberculosis, The 8th Medical Center of PLA General Hospital, Beijing, China; ^2^Department of Tuberculosis, Senior Department of Tuberculosis, The 8th Medical Center of PLA General Hospital, Beijing, China

**Keywords:** tuberculosis, compounds, immunomodulators, immunotherapy, hostdirected therapy

## Abstract

Tuberculosis (TB) is a chronic bacterial disease, as well as a complex immune disease. The occurrence, development, and prognosis of TB are not only related to the pathogenicity of *Mycobacterium tuberculosis* (*Mtb*), but also related to the patient’s own immune state. The research and development of immunotherapy drugs can effectively regulate the body’s anti-TB immune responses, inhibit or eliminate *Mtb*, alleviate pathological damage, and facilitate rehabilitation. This paper reviews the research progress of immunotherapeutic compounds for TB, including immunoregulatory compounds and repurposing drugs, and points out the existing problems and future research directions, which lays the foundation for studying new agents for host-directed therapies of TB.

## 1 Introduction

Tuberculosis (TB) is a chronic respiratory infectious disease caused by *Mycobacterium tuberculosis* (*Mtb*), which remains a major global public health issue. The World Health Organization (WHO) estimates that there were 10.6 million new cases and 1.3 million death cases of TB in 2022 (WHO, 2023). At present, a combination of anti-TB drugs is still the mainstay of TB treatment in clinical practice. Although the standard chemotherapeutic regimens have a satisfactory effect on most drug-sensitive TB, the disadvantages of long treatment periods (at least 6 months), drug toxicity, and poor patient compliance are likely to lead to the emergence of drug-resistant TB, especially multidrug-resistant TB (MDR-TB) or extensively drug-resistant tuberculosis (XDR-TB), which makes the clinical treatment of TB face great challenges (Mi et al., 2021).

**Figure 1:**
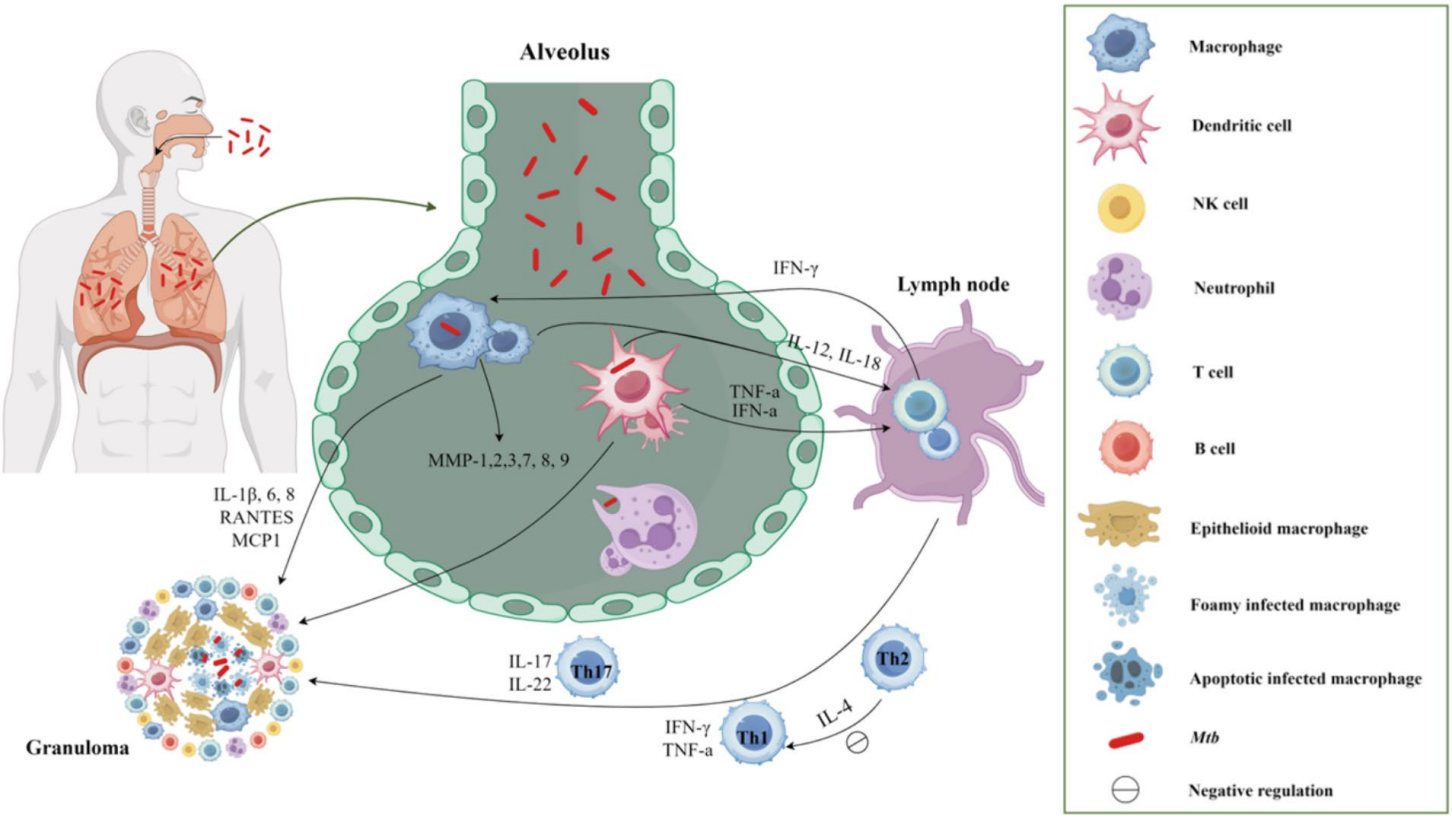
The host′s immune response to TB after *Mtb* infection. Upon entering the respiratory tract and subsequently the alveolus, *Mtb* elicits an immune response in the body. Initially, macrophages and neutrophils serve as the primary defense mechanism, promptly identifying and engulfing *Mtb* through phagocytosis. Following this, macrophages and dendritic cells that have engulfed *Mtb* migrate to the lung parenchyma, leading to the formation of inflammatory lesions and the recruitment of various cell types to aggregate and form granulomas. Moreover, these cells function as classical antigen-presenting cells, releasing IL-12 and IL-18 to facilitate T cell proliferation. Following the initiation of adaptive immunity, the predominant involvement of Th1 cells in the cellular immune response was observed. The secretion of IFN-γ and TNF-α by Th1 cells, along with the secretion of IL-17 by Th17 cells, promptly converged at the infection site, thereby facilitating the formation of granuloma.

Host’s immune responses against TB result from an intricate interplay between the innate and adaptive immune systems (Figure 1), and are determined by the host’s genetic susceptibility, bacterial virulence, and environmental factors. The current treatment regimens mainly focus on various targets of *Mtb* and reduce the bacterial load, but cannot directly weaken the host immunopathological changes associated with TB (Elkington et al., 2011; Hortle and Oehlers, 2020). Enhancing the host’s protective immunity or regulating his anti-TB immune responses maybe a valuable adjunctive therapy for TB, known as host-directed therapy (HDT). HDT agents can help TB patients to improve clinical chemotherapy effects from the following aspects: (1) Regulate the immune status of *Mtb*-infected individuals, improve body anti-TB immunity, enhance the ability to inhibit or eliminate *Mtb*, to prevent the reactivation of latent tuberculosis infection (LTBI) and reduce the incidence of disease; (2) Enhance the therapeutic efficacy of anti-TB drugs in the treatment of TB, especially MDR-TB, shorten the treatment duration, avoid transmission, reduce lung injury, and increase the cure rate; (3) Eliminate the bacteria in phagocytes and reduce the recurrence rate or the risk of reinfection. At present, HDT agents mainly include chemical synthetic drugs, immune system products, and drugs derived from microorganisms. They can regulate the anti-TB immune responses of the body, improve the host’s anti-TB immune function, inhibit or eliminate *Mtb*, reduce pathological damage, and promote recovery. This paper reviews the recent advances in adjuvant therapeutic compounds for TB, including immunoregulatory compounds and repurposing drugs, and points out the existing problems and future research directions, which lays the foundation for studying new agents for TB HDT.

## 2 Immunologically active compounds

It is well known that the discovery of more valuable lead compounds in the early stages of drug development can significantly save effort, money, and time in subsequent drug development. In recent years, a growing number of immunologically active compounds have been found to have a positive adjuvant therapeutic effect in the treatment of TB, including natural products, synthetic compounds obtained through high-throughput screening, computer-aided design, and structural modification. Most of these lead compounds are still in preclinical studies, of which Chicoric acid and CC11050 have entered clinical trials (Table 1).

**Table 1:** Research progress of immunoenhancers in adjuvant therapy of anti-TB.

Compounds/Drugs^a^	Structure	Anti-TB mechanism^b^	Subjects^c^	Number	Phase	CTR number^d^	Status	References
Allicin	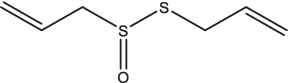	1. Induce protective Th1 response, inhibit the phosphorylation of p38-MAPK, reduce the levels of TNF-α and IL-10; 2. Activate the MAPK and SAPK/JNK pathways, promote IL-1β and IL-12 secretion.	–	–	–	–	–	Dwivedi et al. (2019)
UA and OA	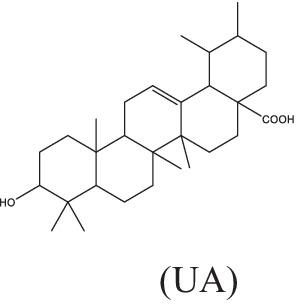 (UA)	1. Stimulate the production of NO and reactive oxygen species, induce the expressions of TNF-α, IL-1β and IL-6, and inhibit TGF-β secretion; 2. Be recognized by CD36 and TGR5, inhibit the NF-κB activation and decrease the secretion of pro-inflammatory cytokines.	–	–	–	–	–	Jiménez-Arellanes et al. (2013), López-García et al. (2015), and Zerin et al. (2016)
	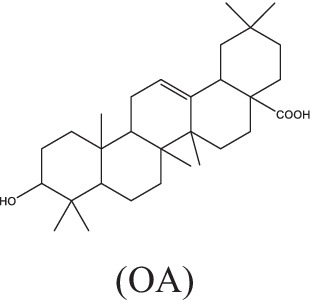 (OA)							
Curcumin	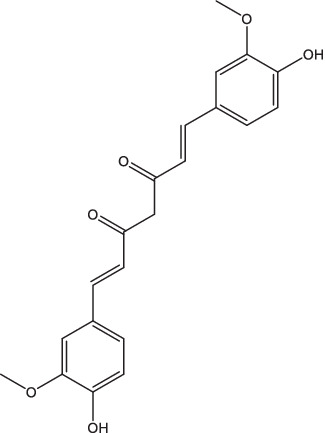	1. Induce caspase-3 dependent apoptosis and autophagy, inhibit NF-κB activation; 2. Induce cathelicidin antimicrobial peptide gene expression.	–	–	–	–	–	Guo et al. (2013), Jäger et al. (2014), and Bai et al. (2016)
[6]-Gingerol	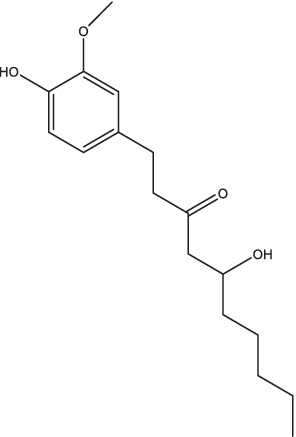	1. Up-regulated expression of pro-inflammatory cytokines, enhance Th1/Th17 response; 2. Increase IFN-γ and IL-17 secretion by CD4^+^ and CD8^+^ T cells; 3. Against both dormant and drug-resistant *Mtb*.	–	–	–	–	–	Bhaskar et al. (2020)
Quercetin-Polyvinylpyrrolidone	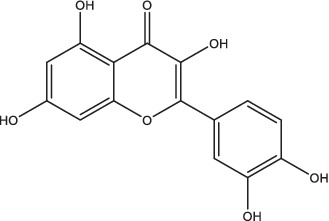	1. Promote IL-4 secretion, decrease IL-1β and TNF-α expression, reduce reactive oxygen species production, prevent caseous necrosis; 2. Improve clinical symptoms and sputum bacteria, promote cavity healing, have favorable safety.	–	–	–	–	–	Butov et al. (2015) and Butov et al. (2016)
Bergenin	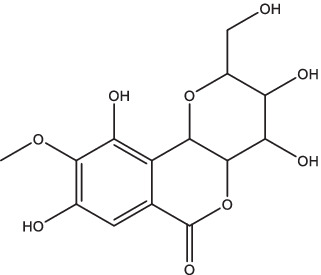	1. Activate MAPK, ERK1/2, and SAPK/JNK pathways; 2. Induce Th1 and Th17 immune responses, promote the secretion of IFN-γ, TNF-α, IL-12 and IL-17; 3. Produce a long-lasting, antigen-specific memory T cells response, reduce the immune damage, bacterial load and the treatment duration of mice.	–	–	–	–	–	Dwivedi et al. (2017) and Kumar et al. (2019)
Chicoric acid	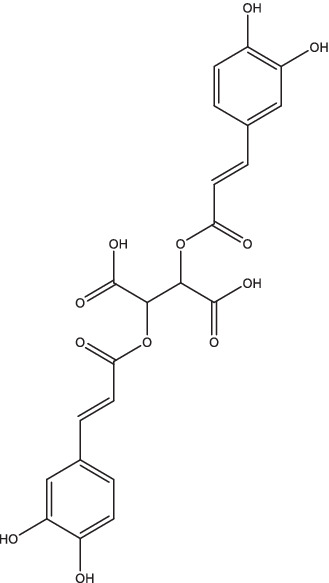	1. Enhance the expression of HLA-DR and CD14 molecules on the surface of *Mtb*-infected U937 macrophages, increase the NO production; 2. Induce phagocytosis and promote the cytokines production.	Patients with MDR-TB; Patients with both COVID-19 and MDR-TB	250	II	NCT05077813	Unknown	Goel et al. (2002), Cundell et al. (2003), and Abd-Nikfarjam et al. (2018)
Luteolin	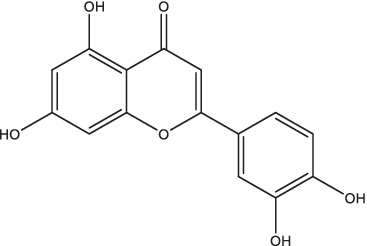	1. Promote NK and NKT cells activity, induce the expression of TCM cells to produce Th1 and Th17 immune responses through inhibiting Kv1.3 K+ channels.	–	–	–	–	–	Singh et al. (2021)
Berberine	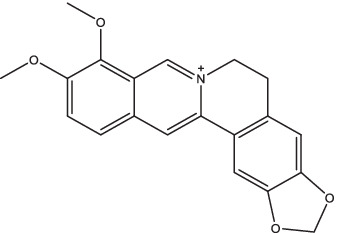	1. Activate the anti-TB activity of macrophages, decrease the expression of pro-inflammatory factors and inhibit NF-κB and JNK kinases; 2. Activate spleen macrophages, increase the B lymphocyte numbers, improve lung pathology.	–	–	–	–	–	Ozturk et al. (2021)
PIMs	–	1. Modulate macrophage activity, act as a ligand for toll-like receptors, C-type lectins, and DC-SIGN to promote early fusion of endosomes; 2. Can be recognized by T cells in the presence of CD1d, activate NKT cell; 3. Bind to α5β1 integrin VLA-5 on CD4^+^ T cells, promote granuloma formation.	–	–	–	–	–	Rojas et al., 2006 and Torrelles et al. (2006)
HE2000	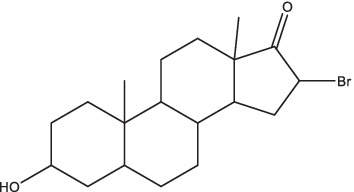	1. Increase the secretion of pro-inflammatory cytokines, reduce the expression level of IL-4; 2. Reduce the expression of 11-βHSD1 and local corticosterone synthesis in mice, activate CD4^+^ Th1 cells and macrophages, promote the expression of protective cytokines.	–	–	–	–	–	Hernandez-Pando et al. (1998), Hernández-Pando et al. (2005), and López-Torres et al. (2021)
CC11050	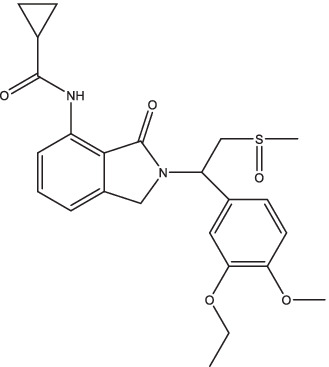	1. Decrease the expression of MMP-1, −12, −14 and genes associated with TNF-α regulation, macrophage activation, and lung inflammation; 2. Combined with INH treatment can reduce the range of lung lesions and improve lung fibrosis.	Smear-positive PTB patients	200	II	NCT02968927	Unknown	Subbian et al. (2016a,b)
Vitamin D	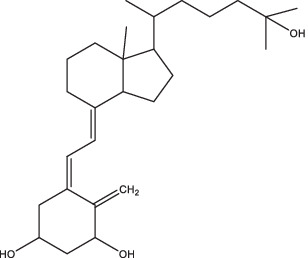 (1, 25-dihydroxy vitamin D3)	1. Inhibit the secretion of pro-inflammatory cytokines; 2. Stimulate the receptor-induced expression of antimicrobial peptides, up-regulate LL37 gene to induce the autophagy related proteins Atg5 and Beclin-1 to mediate the killing effect; 3. Reduce the expression of MMP-7, −9 and − 10, eliminate tissue damage and relieve symptoms of infection; 4. Activate the receptor of vitamin D, reduce *Mtb*-induced bone destruction by inhibiting NF-κB signaling.	Spondylitis TB patients	37	II, III	NCT05376189	Unknown	Martineau et al. (2007), Coussens et al. (2009), Periyasamy et al. (2020), and Deng et al. (2021)
Vitamin D			Children with PTB and vitamin D insufficiency	84	Not Applicable	NCT05073965	Completed	
Levamisole	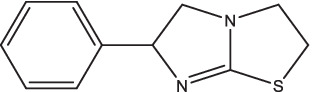	1. Induce the secretion of IL-18, enhance the Th1 immune response; 2. Ameliorate the clinical symptoms, promote the absorption of lesions and negative conversion of sputum bacteria.	–	–	–	–	–	Szeto et al. (2000)
Doxycycline	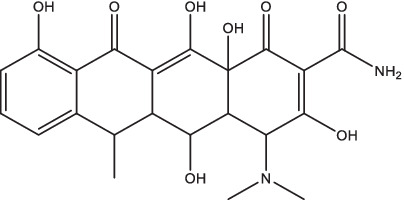	1. Inhibit the secretion of MMP-1 and − 3, down-regulate type I/II interferon and innate immune response genes, up-regulate B cell related genes; 2. Inhibited the growth and activity of *Mtb* containing teT-off promoter-related genes in TB mice; 3. Reduce the activity of type 1 collagenase and elastin in the sputum of TB patients.	PTB patients	40	II	NCT02774993	Completed	Gonzalo et al. (2015), Gengenbacher et al. (2020), Miow et al. (2021), and Simmons and Hawn (2021)
			PTB patients	150	III	NCT05473520	Recruiting	
Aspirin	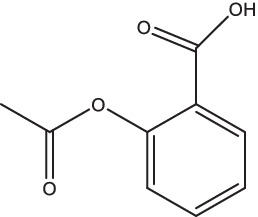	1. Inhibit the cyclo-oxygenase to reduce the production of proinflammatory substances and immunosuppressive substances, alleviate the pathological changes and chronic inflammation, improve the immune response.	TBM patients	768	III	NCT04145258	Recruiting	Maitra et al. (2016) and Young et al. (2020)
Aspirin			Drug-sensitive and MDR-TB patients	354	II	NCT04575519	Recruiting	
Aspirin			TBM patients	327	III	NCT05917340	Not yet recruiting	
Ibuprofen	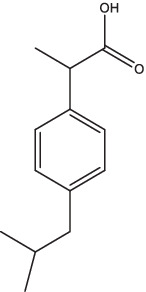		Drug-sensitive and MDR-TB patients	354	II	NCT04575519	Recruiting	
Ibuprofen			XDR-TB patients	24	II	NCT02781909	Completed	
Etoricoxib	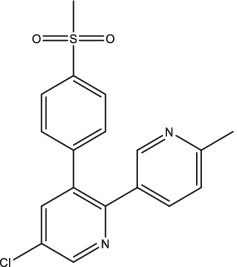		MDR-TB patients	39	I	NCT02503839	Completed	
Celecoxib	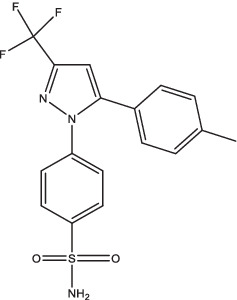		Healthy volunteers	18	I	NCT02602509	Completed	
Pravastatin	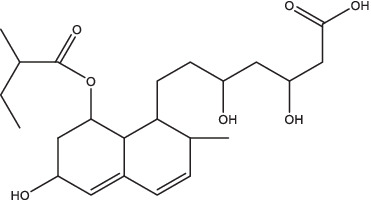	1. Inhibit phagosome acidification and protease hydrolysis; 2. Increase apoptosis, autophagy, cytokine release and NKT cell generation, enhance the anti-TB activity of first-line anti-TB drugs; 3. Regulate Th1 and Th2 cytokine responses, induce the release of pro-inflammatory cytokines, and promote the activation of autophagy and apoptosis.	TB patients	16	II	NCT03882177	Completed	Montero et al. (2000), Dutta et al. (2016, 2020), and Guerra-De-Blas et al. (2019)
Pravastatin			TB patients	16	II	NCT03456102	Completed	
Atorvastatin	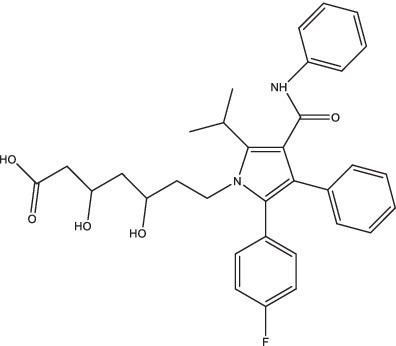		TB patients	440	II	NCT06199921	Recruiting	
Atorvastatin			Cured TB patients with/without HIV infection	220	II, III	NCT04147286	Recruiting	
Rosuvastatin	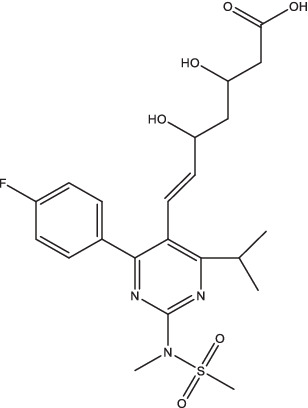		RIF-susceptible TB patients	154	II	NCT04504851	Unknown	
Metformin	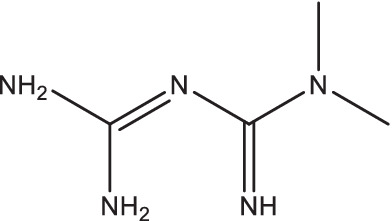	1. Increase the production of mROS in host cells and the acidification of mycobacterium phagosome,;promote the proliferation of T cells secreting IFN-γ; 2. Reduce the soluble CD14, CD163, CRP, and MMP-1, −2, −3, −7, −9, −12 levels; 3. Induce autophagy, regulate oxidative stress, and enhance the efficacy of anti-TB drugs.	PTB patients with HIV	112	II	NCT04930744	Recruiting	Singhal et al. (2014), Kumar et al. (2018), Kumar N. P. et al. (2019), and Fatima et al. (2021)
Loperamide	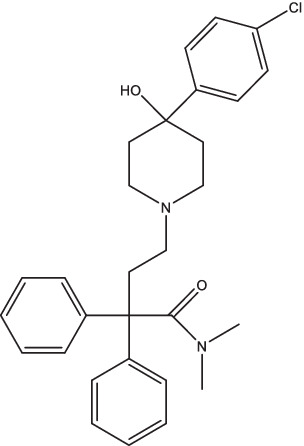	1. Inhibit the growth of *Mtb in vitro*, and regulate the expression of autophagy related genes *in vivo*; 2. Induce the overexpression of bactericidal/permeability increasing protein and antimicrobial peptide LL37 genes; 3. Block the calcium channels to reduce the production of pro-inflammatory cytokines; 4. Induced the production of μ-opioid receptor-dependent antimicrobial peptides, decrease the TNF-α level and increase the production of IL-10 and prostaglandin E2.	–	–	–	–	–	Wetzel et al. (2000), Happel et al. (2011), Ye et al. (2012), Juárez et al. (2016, 2018), and Barrientos et al. (2021)
SASP	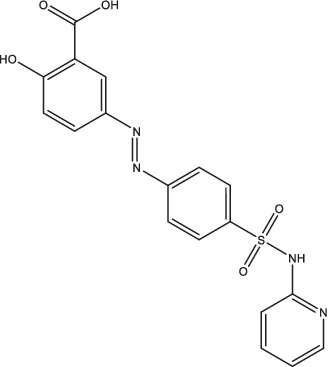	1. Promote the oxidation of mycothiol in *Mtb* macrophages by activating NADPH oxidase to clear *Mtb*; 2. Reduce the *Mtb* bactericidal load and histopathological inflammation of lungs in mice; 3. Prevent severe inflammation induced by *Mtb* infection.	MDR-TB patients	198	N/A	ChiCTR2100045930	Recruiting	Cai et al. (2016), Kang et al. (2017), and Wang W. et al. (2022)
SASP			MDR-TB patients	50	N/A	ChiCTR2000032298	Recruiting	
Dexamethasone	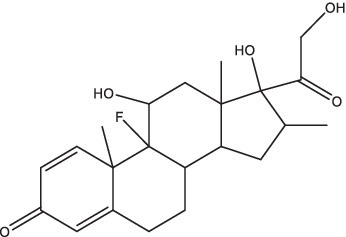	1. Reduce leakage and inflammatory cell infiltration, stabilize lysosomal membrane, relieve bronchospasm, improve pulmonary ventilation; 2. Reduce the local lesion of tuberculous immune pathological injury and serous effusion, and reduce mortality.	HIV co-infected TBM patients	520	III	NCT03092817	Completed	Donovan et al. (2023)
Dexamethasone			TBM patients	720	III	NCT03100786	Active, not recruiting	
Prednisolone	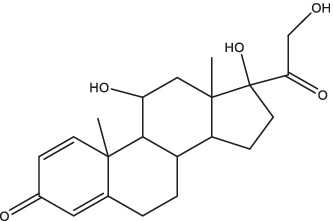		Tuberculous lymphadenitis patients	50	Not Applicable	NCT05861440	Recruiting	
Thalidomide	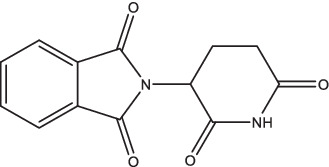	1. Inhibit the TNF-α level, elevate the IFN-γ level, and regulate the NF-κB, IL-6, and IL-1 secretion; 2. Synergistically stimulate CD8^+^ T cells more than CD4^+^ T cells.	Patients complicated tubercular infection of central nervous system	126	II/III	CTRI/2023/05/052417	Recruiting	Panda et al. (2021) and Van Toorn et al. (2021b)
Everolimus	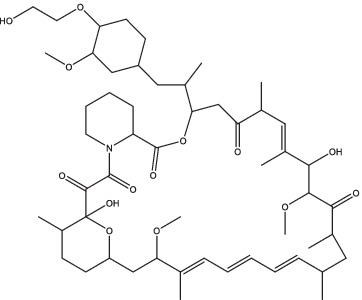	1. Inhibit the mTOR pathway and promote autophagy, increase the ability of macrophages to fight *Mtb*.	Smear-positive PTB patients	200	II	NCT02968927	Unknown	Cerni et al. (2019)
Imatinib	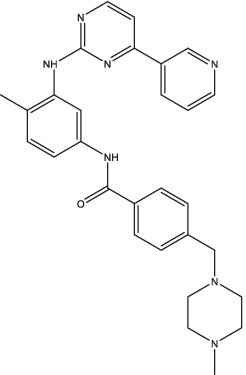	1. Promote autophagy and acidification, enhance the anti-TB effect of RIF, reduce the survival of *Mtb* in macrophages.	TB patients	24	II	NCT03891901	Completed	Napier et al. (2011)
Saquinavir	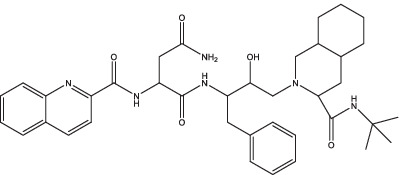	1. Improve the expression of HLA class II antigen, induce the proliferation of CD4^+^ T cells, promote the secretion of IFN-γ.	–	–	–	–	–	Pires et al. (2021)
Zileuton	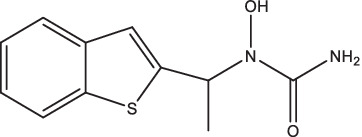	1. Inhibit the production of pro-inflammatory leukotriene and increase the level of IL-1β.	–	–	–	–	–	Mayer-Barber et al. (2014) and Mishra et al. (2020)

^a^UA, Ursolic acid; OA, Oleanolic acid; PIMs, Phosphatidyl-myo-inositol mannosides; SASP, Salazosulfapyridine. ^b^*Mtb*, *Mycobacterium tuberculosis*; INH, Isoniazid; RIF, Rifampicin. ^c^TB, Tuberculosis; MDR-TB, Multidrug-Resistant Tuberculosis; COVID-19, Coronavirus Disease 2019; PTB, Pulmonary Tuberculosis; TBM, Tuberculous Meningiti. ^d^CTR number, Clinical trial registration number; NCT number, ClinicalTrials.gov Identifier; ChiCTR, Chinese Clinical Trial Registry; CTRI, Clinical Trials Registry–India. Only the latest clinical trials are listed here.

### 2.1 Phytochemical compounds

#### 2.1.1 Allicin

Allicin is a trithioallyl ether compound that naturally exists in the bulbs of garlic (*Allium sativum*). Rao et al. first discovered that allicin inhibited the growth of *Mtb* at higher concentrations (Raghunandana Rao et al., 1942). Further studies also have shown that allicin had significant antibacterial effects on drug-sensitive, MDR- and XDR-*Mtb in vitro* and *in vivo*, significantly reducing the bacterial load in mouse drug-sensitive and drug-resistant TB models. In addition, allicin also has good immunoregulatory activity, which can activate the MAPK and SAPK/JNK pathways of *Mtb*-infected mouse peritoneal macrophages to produce various effector molecules, especially inducing an increase in IL-1β and IL-12; can selectively induce strong protective Th1-type responses in murine models; and inhibit the phosphorylation of p38-MAPK to reduce the secretion levels of TNF-α and IL-10 (Dwivedi et al., 2019). In the early stages of human monocytes *Mtb*-infected, allicin could down-regulate the expression of *Mtb ag85b* gene and protein and inhibit the production of reactive oxygen species (ROS), which are through enhancing glutathione peroxidase activity, up-regulating IFN-γ, and inhibiting TNF-α by allicin (Hasan et al., 2006, 2007), indicating that allicin has anti-inflammatory and antioxidant effects. Clinically, the intravenous infusion of allicin as an adjuvant treatment for active pulmonary TB (PTB) patients can improve the symptoms, promote the absorption of the lesions, reduce the secretion of serum inflammatory factors, and improve the effect of chemotherapy, with fewer adverse reactions (Duan, 2014). However, the efficacy of allicin as an adjuvant treatment for TB still needs further clinical verification. Although allicin has anti-TB and immunomodulatory activities, its low water solubility and poor stability greatly limit its biological efficacy. The application of new delivery systems (such as nanoparticles, gels, and liposomes) may improve the stability, encapsulation efficiency, and bioavailability of allicin (Deng et al., 2023).

#### 2.1.2 Ursolic acid and oleanolic acid

Ursolic acid (3β-hydroxyurs-12-en-28-oic, UA) and its isomer oleanolic acid (3β-hydroxyolean-12-en-28-oic, OA) are ubiquitous pentacyclic triterpenoid compounds in food, medicinal herbs, and other plants. Various biological activities have been reported for these two compounds, including antibacterial, antiviral, antiparasitic, antioxidant, anticancer, anti-inflammatory, hepatoprotective, and gastric-protective effects (Namdeo et al., 2023). Gu JQ first reported the anti-*Mtb* effect of UA and OA in *Valeriana laxiflora* and *Quinchamalium majus* in 2004 (Gu et al., 2004; Gua et al., 2004). In recent years, researchers have further demonstrated that UA and OA had *in vitro* activity against mycobacteria (such as drug-sensitive or drug-resistant *Mtb* strains, and a group of non-tuberculosis mycobacterium), with minimum inhibitory concentrations (MICs) ranging from approximately 12.5–100 μg/mL, and had synergistic effects of UA/OA mixtures or with anti-TB drugs (Pitaloka and Sukandar, 2017). UA and OA can significantly reduce bacterial load and lung inflammation in mouse models infected with *Mtb* H_37_Rv or MDR-*Mtb* through subcutaneous or intragastrical administration (Jiménez-Arellanes et al., 2013; López-García et al., 2015). In addition, UA and OA also have good immune regulatory activity. In the early stage of *Mtb*-infected models, treatments with a low dose (0.625 μg/mL) of UA and OA could activate nuclear factor kappa-B (NF-κB), enhance the production of NO and ROS, induce high expression of inducible nitric oxide synthase (iNOS), IFN-γ and TNF-α, and inhibit the expression of TGF-β in mouse macrophages, thereby activating macrophages from M2 phase to M1 phase, inhibiting or killing intracellular *Mtb* (López-García et al., 2015; Podder et al., 2015). UA and OA could be recognized by two cell membrane receptors, CD36 and TGR5, respectively, which could induce overexpression of CD36 and TGR5 receptors. In macrophages and dendritic cells, CD36 acts as a co-receptor for TLR receptors, activating TLR2/1, TLR2/6, or TLR4/6 heterodimers, leading to activation of NF-κB and increase of pro-inflammatory cytokines such as TNF-α, IL-6, and IL-1β secretion (Jiménez-Arellanes et al., 2013; López-García et al., 2015). However, differences in the immune regulatory activity of UA and OA have been observed. Depending on the differences in UA and OA concentration and treatment timing, types and biological status of target cells in the experimental system, they may exhibit anti-inflammatory or pro-inflammatory effects (Ikeda et al., 2008). For example, Zerin et al. (2015, 2016) found that treatment with UA alone significantly inhibited the mRNA expression levels of cytokines (TNF-α, IL-1β and IL-6) and immune regulatory mediators [including iNOS and cyclooxygenase-2 (COX-2)], as well as the release of NO in *Mtb*-infected RAW 267.4 cells, A549 cells, and rat splenocytes stimulated by Con A. From this, it can be seen that using UA for anti-inflammatory treatment may be a double-edged sword. Further evaluation is needed to assess the effects of UA dosage and treatment timing on the biological status of target cells or tissues. Besides, a recent study showed that UA could synergically inhibit Akt/mTOR and TNF-α/TNFR1 signaling pathways, and promote autophagy, thereby inhibiting the pyroptosis and necroptosis of macrophages induced by *Mtb*, and enhancing the intracellular killing of *Mtb* (Shen et al., 2023). Although the combination of antibacterial and immunomodulatory activities of UA and OA may make them potential agents for TB adjuvant treatment, it is worth noting that high doses of UA and OA (25–50 μg/mL) are highly toxic to macrophages, the poor penetration, low lung concentration, and severe side effects caused by subcutaneous and oral administration of UA and OA may hinder the clinical application of these compounds to some extent. Saini et al. directly administered UA and OA to the lungs of Wistar rats using a metered dose inhaler (MDI). Pharmacokinetic results showed that the combined therapy could increase the biological half-life of UA and OA, and the drug concentration in the lungs was higher than in the systemic circulation. Acute inhalation toxicity studies also showed good safety and no adverse effects on body weight and important organs (Saini et al., 2022).

#### 2.1.3 Curcumin

Curcumin is a natural hydrophobic polyphenol substance extracted from the roots and stems of some plants in the ginger and Araceae families. Schraufstatter and Bernt first discovered that curcumin diluted at 1:40000 showed a significant inhibitory effect on *Mtb* in 1946 (Schraufstatter and Bernt, 1949). Further studies have shown that curcumin could induce caspase-3-dependent apoptosis and autophagy, inhibit NF-κB activation, and protect macrophages from P19-induced apoptosis to kill intracellular *Mtb* in macrophages (Zhang et al., 2014; Bai et al., 2016; Ansari et al., 2023). In addition, curcumin could induce the expression of cathelicidin antimicrobial peptide gene through a vitamin D receptor-independent pathway, which may contribute to the clearance of *Mtb* in the human body (Guo et al., 2013). Animal experiments have shown that intraperitoneal injection of curcumin could reduce the lung bacterial load and pneumonic area in *Mtb* H_37_Rv-infected BALB/c mouse models, reduce neuroinflammation, and improve behavioral status (Lara-Espinosa et al., 2022). Besides, previous studies suggested curcumin had a protective effect on INH-induced liver injury and could significantly reduce the abnormal increase of liver function biomarkers (He et al., 2017; Li et al., 2022). A study conducted in India has demonstrated that curcumin could significantly elevate the blood concentration of Bedaquiline by limiting P-glycoprotein-mediated efflux to improve the absorption and slow the metabolism of Bedaquiline, so it is advised not to combine curcumin with Bedaquiline to prevent potential adverse reactions (Kotwal et al., 2020). However, poor oral availability, limited gastrointestinal absorption, and fast metabolism have been the obstacles to the clinical application of curcumin (Hussain et al., 2022). Recent studies have shown that the use of nanotechnology to encapsulate curcumin may help address this problem. Curcumin nanoparticles have been shown to stimulate innate immune responses, promote autophagy in antigen-presenting cells (APCs), and stimulate the production of co-stimulatory molecules and pro-inflammatory cytokines in APCs. Furthermore, curcumin nanoparticles have also been found to enhance the ability of the Bacillus Calmette-Guérin (BCG) vaccine to induce central memory T cells (T_CM_) of the Th1 and Th17 lineages, potentially improving the efficacy of the BCG vaccine (Ahmad et al., 2019). And other studies have also found that the nanoparticle-encapsulated curcumin could dramatically increase the bioavailability of curcumin in animals, and the adjuvant treatment with nano-curcumin in combination with INH or RIF revealed a satisfactory bactericidal effect, hepatotoxicity reduction, a shorter treatment time, and low risk of disease recurrence (Tousif et al., 2017; Galdopórpora et al., 2022; Li et al., 2022; Gupta et al., 2023). Therefore, whether curcumin can be used as a therapeutic adjuvant for TB needs to be further confirmed in animal models and clinical trials.

#### 2.1.4 [6]-Gingerol

[6]-Gingerol is the main active ingredient of ginger (*Zingiber officinale*), which has anti-inflammatory, antioxidant, anticancer, and immunomodulatory functions (Wang et al., 2014). It was found that [6]-gingerol could effectively inhibit the growth of *Mtb* in the lung, spleen, and liver of *Mtb*-infected mice (Bhaskar et al., 2020). And [6]-gingerol-intraperitoneally injected mice showed a significantly up-regulated expression of pro-inflammatory cytokines in the spleen, enhanced Th1/Th17 response, and increased IFN-γ and IL-17 secretion by CD4^+^ and CD8^+^ T cells (Bhaskar et al., 2020). Besides, bacterial load in the lungs was significantly reduced in mice when [6]-gingerol (intraperitoneal injection) was used in combination with INH (Bhaskar et al., 2020). In addition, [6]-gingerol has been found to possess great anti-TB potency against both dormant *Mtb* and drug-resistant *Mtb* (Bhaskar et al., 2020). The pharmacokinetic results showed that the maximum blood concentration (C_max_) and time to reach C_max_ (T_max_) of 6-gingerol were 453.40 ng/mL and 30 min. The elimination half-life (t_1/2_) is 149 min (Levita et al., 2018). Another pharmacokinetic study has indicated that [6]-gingerol may increase the oral bioavailability of Bedaquiline by modifying gastrointestinal physiology, potentially leading to adverse reactions or side effects (Kotwal et al., 2020). [6]-gingerol, as a new adjuvant anti-TB and immunomodulatory candidate, has a good prospect in the treatment of sensitive- and resistant-*Mtb* strains, but there is still a lack of critical experimental data to guarantee its quality, efficacy, and safety.

#### 2.1.5 Quercetin-polyvinylpyrrolidone

Many flavonoids have been described as having antimycobacterial activity (Sharma and Yadav, 2017). Quercetin, a kind of flavonoid from natural plants, shows anti-inflammatory and antioxidant effects but possesses poor solubility and low bioavailability. Quercetin coupled with Polyvinylpyrrolidone (QP) could effectively improve its solubility and has immunomodulatory activity to promote the growth of endothelial cells and reduce inflammation and blood coagulation (Butov et al., 2016). *In vitro* experiments showed that quercetin possessed 99.3% inhibitory activity against *Mtb* H_37_Rv at 200 μg/mL, with a MIC value of 6.25 μg/mL. It also had a strong binding affinity with the N-terminus of isocitrate lyase (ICL), with an IC_50_ of 3.57 μM. These data strongly suggest an inhibition activity of quercetin on *Mtb* (Shukla et al., 2015; Sasikumar et al., 2018). Dmytro et al. have shown that QP could promote IL-4 secretion by helper T cells, decrease IL-1β and TNF-α expression, reduce production of reactive oxygen species by macrophages, and prevent caseous necrosis (Butov et al., 2016). Animal experiments have demonstrated that intraperitoneal injection or intragastric administration QP can limit the extent of pathological progress and tissue necrosis, protect hepatocytes, and repair infected lung tissues (Butov et al., 2015; Zhang et al., 2020; Sanjay et al., 2021). After the treatment of active PTB with QP (intravenous injection) combined with anti-TB drugs, the patients revealed a reduction in clinical symptoms and sputum bacteria, marked absorption of lung lesions, a shorter cavity closure, and a favorable safety profile, which suggests that QP may be offered as a potential candidate for TB immunotherapy (Butov et al., 2016). Currently, 3D-printed pharmaceutical skin patches containing QP have been developed to provide appropriate therapeutic drug concentration and satisfactory sustained-release characteristics and may be used for the treatment of TB patients in the future (Chaudhari et al., 2021).

#### 2.1.6 Bergenin

Bergenin, a type of polyphenol compound isolated from plants in the genus *Bergenia*, exhibits antitussive, analgesic, and anti-inflammatory properties. Studies found that Bergenin had anti-mycobacterial activity through screening and further demonstrated that although bergenin is unable to kill *Mtb* directly, it could activate MAPK, ERK1/2, and SAPK/JNK pathways of *Mtb*-infected macrophages to clear bacteria (Dwivedi et al., 2017; Kumar N. P. et al., 2019). In addition, intraperitoneal injection of bergenin could also selectively induce Th1 and Th17 immune responses in *Mtb*-infected mice, promote the secretion of IFN-γ, TNF-α, IL-12, and IL-17, and induce NO production, which contributes to the inhibition of *Mtb* growth (Dwivedi et al., 2017). A long-lasting, antigen-specific memory T cell response was generated in a TB murine model when bergenin was co-treated with INH, and the immune damage and bacterial load of mice were significantly reduced and the treatment duration was shortened remarkably (Kumar S. et al., 2019). The pharmacokinetic results of a single oral administration (12 mg/kg) of bergenin showed a C_max_ of 957 ng/mL and a t_1/2_ of 0.36 h, indicating that a certain amount of bergenin could be absorbed and rapidly converted to its metabolites (Ren et al., 2019). These studies indicated that bergenin may be a powerful immunomodulator, which may further explore its value as an adjuvant therapy for TB.

#### 2.1.7 Chicoric acid

Chicoric acid is a hydroxycinnamic acid with excellent antioxidant, hepatoprotective, and anti-inflammatory bioactivities. It has been demonstrated *in vitro* that the combination of chicoric acid and 13-*cis*-retinoic acid could enhance the expression of HLA-DR and CD14 molecules on the surface of *Mtb*-infected U937 macrophages, significantly increase the production of NO, and inhibit the growth of *Mtb* in macrophages (Abd-Nikfarjam et al., 2018). Chicoric acid has previously been shown to have immunological activity, inducing phagocytosis, increasing the number of immune cells, and promoting the production of cytokines (Goel et al., 2002; Cundell et al., 2003). The pharmacokinetic results of 50 mg/kg administration of chicoric acid in SD rats showed that the t_1/2_ was 4.53 ± 1.44 h, the mean residual time was 18.58 ± 4.43 h, and the area under the curve (AUC) was 26.14 mg⋅h/L. The concentration distribution of chicoric acid in liver, lung and kidney was higher than that in other tissues (Wang et al., 2016). A double-blinded, randomized, phase II clinical trial is currently underway to evaluate the role of initial combination treatment with retinoic acid, chicoric acid, minocycline, and vitamin D in the treatment of MDR-TB and both MDR-TB and COVID-19 patients (ClinicalTrials.gov Identifier: NCT05077813), but the current status of this study is unknown.

#### 2.1.8 Luteolin

Luteolin is a natural flavonoid widely found in various vegetables, fruits, and medicinal plants, which has anti-inflammatory, anticancer, and antioxidant effects. A previous study indicated, for the first time, the potential anti-mycobacterial activity of luteolin isolated from the flowers of *Chromolaena odorata* (*Eupatorium odoratum*) (Suksamrarn et al., 2004). The *in vitro* experiments confirmed that luteolin has certain anti-TB and hepatoprotective activities (Araujo et al., 2014; Shabbir et al., 2020). Recent studies have found that luteolin (intraperitoneal injection) and INH (oral) combination therapy can promote the activity of natural killer cells and natural killer T cells, induce the expression of type central memory T cells to produce Th1 and Th17 immune responses effectively through inhibiting Kv1.3 K^+^ channels, so as to shorten the treatment duration of TB, alleviate INH-induced hepatotoxicity, prevent the recurrence of disease and the emergence of drug-resistant *Mtb* (Singh et al., 2021). Following oral administration of SD rats with luteolin at the dosage of 200 mg/kg, the pharmacokinetic parameters in rat plasma were obtained with the technology of HPLC–MS/MS, indicating that the t_1/2_ at 4.58 ± 2.55 h, C_max_ at 152.9 ± 35.1 ng/mL, T_max_ at 0.25 ± 0.04 h, and AUC_0–t_ at 682.6 ± 170.6 ng⋅h /mL (Wu et al., 2022). At present, only computerized ADME and toxicity evaluation have confirmed that luteolin is effective and safe for oral administration (Islam et al., 2023), and there is no support from animal experiments or clinical trial data.

#### 2.1.9 Berberine

Berberine, as a representative benzylisoquinoline alkaloid, has shown potent activity against resistant *Mtb* strains since 1998 (Gentry et al., 1998). The available data from *in vitro* experiments indicates that berberine demonstrates immunomodulatory properties by primarily activating macrophages and enhancing their *Mtb*-killing abilities, while simultaneously decreasing the expression of pro-inflammatory factors (Ozturk et al., 2021). This dual effect may be attributed to berberine’s ability to exert anti-TB effects on macrophages through alternative mechanisms, such as inducing autophagy via the AMPK/mTOR pathway and mouse uncoupling protein 2, thereby suppressing inflammation in mouse macrophages (Wang et al., 2011; Fan et al., 2015). A recent study found that berberine enhances the innate defense mechanism against *Mtb* and stimulates differentiation of Th1/Th17 specific effect memory and tissue-resident memory responses through the NOTCH3/PTEN/AKT/FOXO1 pathway, thereby augmenting the host resistance against TB (Pahuja et al., 2023). Oral berberine alone or in combination with RIF and INH could activate spleen macrophages and increase the B lymphocyte numbers, as well as reduce lung pathology but without additive or synergistic effects on bacterial burden in C57BL/6 mice (Ozturk et al., 2021). The pharmacokinetic results of single intravenous administration (4.0 mg/kg) and oral administration (48.2, 120 or 240 mg/kg) of berberine in rats showed that berberine metabolized rapidly *in vivo* with absolute bioavailability of 0.37 ± 0.11%, suggesting that berberine had low absolute bioavailability, insufficient absorption and extensive metabolism (Feng et al., 2020). Thus, overcoming low bioavailability remains a significant challenge. At present, computer screening has found that *Mtb*-FtsZ may be a target for berberine to exert an anti-TB effect as they can form a stable complex coupled with a significantly high binding energy (Akinpelu et al., 2022). Of course, this finding needs to be verified in later cell and animal experiments.

### 2.2 Microbe-derived components

#### 2.2.1 Phosphatidyl-myo-inositol mannosides

Phosphatidyl-myo-inositol mannosides (PIMs) are glycolipids found in the cell envelope of all *Mycobacterium* species with immunomodulatory activities (Boldrin et al., 2021). PIMs could modulate macrophage activity and cytokine production and could be recognized by macrophage mannose receptors, dendritic cell-specific ICAM-3-grabbing nonintegrin, or other receptors depending on the structural differences between PIMs, and this balance may directly influence the immune response of the host to *Mtb* infection (Torrelles et al., 2006). In addition, PIMs also could be recognized by T cells in the presence of CD1d, activate NKT cells to produce specific immune responses, and bind to α_5_β_1_ integrin VLA-5 on CD4^+^ T cells, resulting in T cell adhesion to fibronectin and promoting granuloma formation (Rojas et al., 2006). Several compounds of PIMs and their related components have been synthesized, such as PIM4, ACPIM6, PIM6, and AC2PIM2. More recently, tetraacylated phosphatidylinositol hexamannoside (Ac2PIM6) synthesized from stearic acid and tuberculous stearic acid has been shown to significantly increase serum levels of IL-4 and IFN-γ in mice, and to have certain adjuvant effects on albumin and tetanus toxoid antigens (Patil et al., 2015). However, its immunotherapeutic effect on TB needs to be further studied.

### 2.3 Synthetic compounds

#### 2.3.1 HE2000

HE2000 (16alpha-Bromoepiandrosterone), an androsterone adrenal steroid derivative, exerts effective activity in the recovery of immune responses in a variety of diseases such as HIV, malaria, and TB. Studies have indicated that subcutaneous HE2000 combined with oral chemotherapy can lead to a significant increase in the secretion of pro-inflammatory cytokines (IFN-γ, IL-2, and TNF-α) in lung tissue homogenate of *Mtb*-infected mice. This combination also results in a decrease in the expression level of IL-4, a reduction in bacterial load, a shortened duration of antibiotics treatment, and an improvement in survival rate (Hernandez-Pando et al., 1998; Hernández-Pando et al., 2005). The elevated secretion of pro-inflammatory cytokines may be attributed to the shift in cytokine profile from Th2 to Th1 in mice at advanced stages of infection following HE2000 treatment (Hernández-Pando et al., 2005). It is well known that patients with type 2 diabetes mellitus have increased severity of TB. One of the important pathogenic factors is the high expression of 11-βHSD1 and corticosterone in the lung and liver in advanced disease (López-Torres et al., 2021). Intratracheal or subcutaneous administration of HE2000 could effectively reduce the expression of 11-βHSD1 and local corticosterone synthesis in *Mtb*-infected mice, thereby activating CD4^+^ Th1 cells and macrophages, promoting the high expression of protective cytokines (TNF-α, IFN-γ), and significantly reducing *Mtb* burden and hyperglycemia (Hernández-Pando et al., 2005; López-Torres et al., 2021). Combined with the fact that HE2000 is not metabolized into sex steroids, these findings revealed this safe and well-tolerated compound may be a potential immune modulator for the adjuvant treatment of TB.

#### 2.3.2 CC11050

CC11050, a type 4 phosphodiesterase inhibitor, has been shown in preclinical studies to improve inflammation and injury caused by TB (Subbian et al., 2016a). After 8 weeks gavage treatment with CC11050, the rabbits showed a decreased expression of multiple matrix metalloproteinase genes (MMP-1, −12, −14), as well as other genes associated with TNF-α regulation, macrophage activation, and lung inflammation, thus blocking the mechanism leading to permanent, progressive loss of lung function (Subbian et al., 2016a). CC11050 had no direct anti-TB activity when administered *in vitro* or alone, but combined with INH treatment by oral gavage could significantly reduce the range of lung lesions, improve lung fibrosis, and enhance the osmotic effect of INH in lung lesions in TB animal models (Subbian et al., 2016a,b). Pharmacokinetic experiments revealed that CC11050 was absorbed at a faster and more rapid rate in mice compared to rabbits (T_max_ and C_max_ of 1,410 ng/mL at 2 h, 163 ng/mL at 4 h). When combined with INH, the T_max_ of CC11050 in mice was delayed to 5 h, and there was a moderate increase in plasma C_max_ and AUC_last_. This may be attributed to the inhibitory effect of INH on the metabolism of CC11050. However, this effect was not observed in the co-treated rabbit model (Subbian et al., 2016a,b). An open-label, phase II, randomized controlled trial of CC11050, everolimus, auranofin, and ergocalciferol in PTB patients may be active in South Africa (ClinicalTrials.gov Identifier: NCT02968927). The results of this study showed that CC11050 is safe and fairly well tolerated as an adjunctive treatment for TB, and may also promote recovery of FEV1, a key indicator of lung function (Wallis et al., 2021).

## 3 Repurposed drugs

Many drugs used for the treatment of other diseases are also used to treat TB as adjuncts (Table 1), these agents contribute to the clearance of *Mtb* infection and the prevention of the development of drug resistance when used in conjunction with clinical short-course chemotherapy regimens.

### 3.1 Vitamin D

Vitamin D is a fat-soluble vitamin, while it does not possess any biological activity. It has to be converted to 1, 25-dihydroxyvitamin D3 *in vivo* to facilitate intestinal calcium absorption and prevent nutritional rickets in children. In recent years, several studies have found that vitamin D is involved in the body’s immune response to TB. The *in vitro* experiments have confirmed that vitamin D could inhibit the growth of *Mtb* and secretion of pro-inflammatory cytokines in peripheral blood monocytes (Martineau et al., 2007), stimulate the receptor-induced expression of antimicrobial peptides, and up-regulate LL37 gene to induce the autophagy-related proteins Atg5 and Beclin-1 to mediate the killing effect (Periyasamy et al., 2020). In addition, it could also reduce the expression of MMP-7, MMP-9, and MMP-10 in human peripheral blood monocytes, and increase the expression of TIMP-1, a tissue inhibitor of MMP, to eliminate tissue damage and relieve symptoms of infection (Coussens et al., 2009). Animal models suggested that activation of vitamin D receptor reduced *Mtb*-induced bone destruction by inhibiting NF-κB signaling (Deng et al., 2021). Furthermore, it has been shown that oral vitamin D combined with PZA decreased the release of inflammatory cytokines and increased the expression of anti-microbial peptides (Zhang et al., 2019). After treatment with INH, RIF, and pyrazinamide alone or in conjunction with immunomodulator drugs (such as all-*trans* retinoic acid, vitamin D3, and α-galactose amide), a notable decrease in *Mtb* burden and TB recurrence rate, and a shortened treatment course were observed in PTB mice (Mourik et al., 2017). Additionally, oral vitamin D supplements were found to expedite sputum smear conversion in smear-positive PTB patients and hasten the resolution of inflammatory responses linked to a heightened mortality risk. Furthermore, it was noted that vitamin D supplements could aid in the resolution of treatment-induced lymphopenia, monocytosis, hypercytokinaemia, and hyperchemokinaemia (Coussens et al., 2012). At present, there are several clinical studies on TB patients with vitamin D-assisted chemotherapy, but the therapeutic effect on drug-sensitive TB patients remains unsatisfactory, while the sputum-negative conversion rate of a few MDR-TB patients seems to be significantly improved (Wallis and Zumla, 2016). However, Bekele et al. found that the combination of vitamin D3 and sodium phenylbutyrate with standard anti-TB treatment enhanced the killing effect of macrophages on *Mtb*, and significantly improved the clinical symptoms of patients, but had no effect on sputum clearance rate (ClinicalTrials.gov Identifier: NCT01698476) (Bekele et al., 2018). Vitamin D immunotherapy was generally safe and well tolerated, but a few patients had serious adverse reactions, such as paravertebral abscess enlargement, psoas abscess enlargement, hypercalcemia, etc. (Wallis and Zumla, 2016). In addition, few studies on the pharmacokinetics of vitamin D in animals or humans have been reported. Therefore, it is necessary to establish consistent clinical protocols such as patient population, dosage and duration of treatment in order to explore the safety and efficacy and collect the pharmacokinetic parameters of vitamin D in the treatment of TB more comprehensively. A randomized controlled trial of vitamin D supplementation effect in PTB children with vitamin D insufficiency has been completed in Indonesia (NCT05073965), and the result of this study provided strong evidence that vitamin D improves fever, cough, and nutritional status in these patients (Tamara et al., 2022). Currently, a randomized clinical trial is underway to evaluate the effect of oral vitamin D supplementation on the expression of TLR-2, TLR-4, and clinical outcomes in spondylitis tuberculosis patients and the status of this trial is currently unknown (ClinicalTrials.gov Identifier: NCT05376189).

### 3.2 Levamisole

Levamisole, a synthetic imidazo (2,1-b) thiazole derivative, is known as a broad-spectrum anthelmintic in clinical practice. As early as 1981, levamisole was found to significantly increase the positive rate of dinitrochlorobenzene (DNCB) skin tests in PTB patients, suggesting that levamisole can attenuate immunosuppression after *Mtb* infection (Singh et al., 1981). In recent years, levamisole has been used as an immunomodulator to ameliorate immune damage in autoimmune diseases, cancers, primary immunodeficiency diseases, and infectious diseases. The Brown Norway rats injected intraperitoneally with levamisole showed a marked upregulation of IL-18 and enhancement of Th1 immune response (Szeto et al., 2000). Clinical oral levamisole adjuvant treatment of TB and bone TB patients could ameliorate the clinical symptoms, improve the body’s immune functions, and promote the absorption of lesions and negative conversion of sputum bacteria, but may cause gastrointestinal discomfort, allergy, headache, fever, dizziness and other adverse reactions (Mielants and Veys, 1978; Qiu et al., 2002; Guo, 2008; Shamkuwar et al., 2017; Saurabh et al., 2018). However, detailed pharmacokinetic data from large number of subjects are lacking.

### 3.3 Doxycycline

Doxycycline, a semi-synthetic tetracycline antibiotic, is currently the only licensed broad-spectrum MMP inhibitor. Initially, doxycycline was found to have promising scavenging or inhibition effect toward *Mycobacterium gordonae*, *Mycobacterium fortuitum*, and *Mycobacterium marinum* (Wallace and Wiss, 1981; Tasaka et al., 1995; Weyers et al., 1996). Later, it was discovered that doxycycline has a promising anti-*Mtb* effect. Doxycycline could inhibit the secretion of MMP-1 and MMP-3 in *Mtb*-infected macrophages, down-regulate type I/II interferon and innate immune response genes, and up-regulate B cell biology-related genes (Miow et al., 2021; Simmons and Hawn, 2021).

*In vitro* experiments showed that doxycycline combined with amikacin had either an indifferent or synergistic effect on clinically isolated drug-resistant *Mtb* strains, but had no antagonistic effect (Gonzalo et al., 2015). Gengenbacher M et al. found that adding 2,000 ppm of doxycycline to the standard diet was sufficient to achieve complete inhibition of tet promoters in infected tissues of TB mice and rabbits, thereby affecting the growth and activity of *Mtb* (Gengenbacher et al., 2020). Doxycycline injection for 4 weeks in rabbits revealed a significant reduction in serum MMP-1 levels and blocked the progression of infection to alleviate the destruction of vertebral tissue by tuberculous spondylitis (Siregar et al., 2023).

Doxycycline is rapidly absorbed through the gastrointestinal tract, and its bioavailability is between 75 and 100% (Zhanel et al., 2004). The maximum blood concentration was reached within 4 h, and the half-life was 12 h (Zhanel et al., 2004). Doxycycline penetrates well into tissues and organs due to its lipophilic properties. About 30–40% of doxycycline is excreted intact in the urine (Zhanel et al., 2004). A phase II clinical trial conducted in Singapore showed that doxycycline in combination with anti-TB drugs reduced the activity of type 1 collagenase and elastin in the sputum of TB patients and was well tolerated (ClinicalTrials.gov Identifier: NCT02774993) (Miow et al., 2021). Another phase III clinical trial is being performed by the National University Hospital of Singapore to determine the improvement of lung function and decrease in tissue destruction of doxycycline in PTB patients (ClinicalTrials.gov Identifier: NCT05473520), and this study is recruiting subjects. Doxycycline is safe when taken orally, but the most common side effects are gastrointestinal problems and skin reactions (Smith and Leyden, 2005).

### 3.4 Nonsteroidal anti-inflammatory drugs

It is well known that nonsteroidal anti-inflammatory drugs (NSAIDs) are widely used for antipyretic, analgesic, and anti-inflammatory actions. NSAIDs could reduce the production of proinflammatory substances and immunosuppressive substances by inhibiting the cyclo-oxygenase, to alleviate the pathological changes and chronic inflammation of the host lungs and improve the immune response (Maitra et al., 2016; Young et al., 2020).

A randomized, active-controlled, phase III clinical trial was performed in tuberculous meningitis (TBM) patients to assess the efficacity of aspirin to reduce mortality (ClinicalTrials.gov Identifier: NCT04145258). Another phase II clinical trial is being performed in Germany to assess the efficacy and safety of 2 repurposed drugs (acetylsalicylic acid and ibuprofen) in drug-sensitive and MDR-TB patients (ClinicalTrials.gov Identifier: NCT04575519). These two studies are all currently recruiting participants. Furthermore, a clinical trial of aspirin with or without high-dose RIF, moxifloxacin, INH, Pyrazinamide, and steroids in TBM patients is being performed by the Tuberculosis Research Center of India to comparatively evaluate the short course regimen and standard regimen (ClinicalTrials.gov Identifier: NCT05917340), but this study is not yet recruiting the patients. In 2019, a prospective, randomized, pilot study to estimate the potential efficacy and safety of using adjunctive ibuprofen for the treatment of XDR-TB patients was completed but without results posted (ClinicalTrials.gov Identifier: NCT02781909).

To determine the safety and immunogenicity of etoricoxib, a phase I clinical trial has been completed in MDR-TB patients in Norway (ClinicalTrials.gov Identifier: NCT02503839). The results showed that etoricoxib adjuvant therapy was safe in TB patients, and only 3 patients developed minor adverse reactions (Jenum et al., 2021). Etoricoxib could not improve cellular immunity and humoral immunity but decreased the H56: IC31-induced T cell response (Jenum et al., 2021). Moreover, this study suggested that impaired macrophage capacity in TB patients treated with etoricoxib may have a potentially unfavorable effect on the control of mycobacterial growth (Nore et al., 2023).

A recent study concluded that COX2 inhibitors (acetylsalicylic acid and celecoxib) may downregulate inflammation in TB patients, but further investigations are needed to evaluate the potential mechanisms in a larger clinical trial (Carranza et al., 2023). A study of celecoxib in healthy volunteers to evaluate the bactericidal activity against *Mtb* has been completed (ClinicalTrials.gov Identifier: NCT02602509), but the results showed celecoxib with or without RIF or pyrazinamide have no anti-mycobacterial effect in the whole-blood bactericidal activity model (Naftalin et al., 2018).

### 3.5 Statins

Statins, inhibitors of 3-hydroxy-3-methylglutaryl-coenzyme A reductase, have primarily been utilized for cardiovascular disease and lipid disorders because of their cholesterol-lowering effects.

In recent years, studies have found that statins also have anti-inflammatory, immunomodulatory, and pathogenic bacteria inhibition effects (Xu et al., 2017; Parihar et al., 2019; Woźniak et al., 2023), and there are several researches on the role of statins in adjuvant immunotherapy of TB (Dutta et al., 2020; Hu et al., 2020). Pravastatin could effectively inhibit phagosome acidification and protease hydrolysis, which is conducive to *Mtb* clearance (Dutta et al., 2020). Simvastatin inhibited *Mtb* infection by increasing apoptosis, autophagy, cytokine release, and NKT cell generation, and could assist in enhancing the anti-TB activity of first-line anti-TB drugs and shorten the cure time (Dutta et al., 2016; Guerra-De-Blas et al., 2019). Fluvastatin could regulate Th1 and Th2 cytokine responses, induce the release of pro-inflammatory cytokines IL-1β, IL-18, and IFN-γ, and promote the activation of autophagy and apoptosis (Montero et al., 2000; Dutta et al., 2020). Besides, rosuvastatin could also inhibit the growth of *Mtb* (Dutta et al., 2020).

Recently, two phase II clinical trials to evaluate the safety, tolerability, pharmacokinetics, and efficacy of pravastatin in TB patients have been completed but no results published so far (ClinicalTrials.gov Identifier: NCT03882177 and NCT03456102).

In addition, a phase II, randomized, double-blind clinical trial is being performed by Obafemi Awolowo University Teaching Hospital (Ile Ife, Osun, Nigeria) to evaluate the safety and efficacy of different doses of atorvastatin-containing regimen in TB patients (ClinicalTrials.gov Identifier: NCT06199921). Another clinical trial is also conducting by the University of Cape Town (Observatory, WC, South Africa) to determine the effectiveness of atorvastatin in reducing persistent inflammation after TB treatment completion in HIV-infected and HIV-uninfected patients (ClinicalTrials.gov Identifier: NCT04147286).

Additionally, a phase II clinical trial in PTB patients has been conducted in Singapore to determine whether the addition of rosuvastatin to standard TB therapy results in accelerated sputum culture conversion (ClinicalTrials.gov Identifier: NCT04504851). The study results provided strong evidence that adjunctive rosuvastatin once per day was safe but there were no substantive improvements in the culture conversion (Cross et al., 2023).

### 3.6 Metformin

Metformin is a biguanide drug widely used for patients with type 2 diabetes worldwide. Diabetes increases susceptibility to TB by indirectly affecting the functions of macrophages and lymphocytes, such as chemotaxis, phagocytosis, and antigen presentation (Kumari and Meena, 2014). Metformin could increase host cells production of mitochondrial ROS and the acidification of mycobacterium phagosome, promote the proliferation of T cells secreting IFN-γ, reduce the bacterial load, TB-induced tissue pathology and inflammatory response in both lung and spleen of mice, and enhance the host-specific immune function (Singhal et al., 2014). Secondly, metformin treatment reduced soluble CD14, CD163, and inflammatory C-reactive protein levels in TB patients with diabetes, significantly decreased MMP-1, −2, −3, −7, −9, and − 12 levels, and promoted smear conversion, indicating that metformin has significant anti-inflammatory and lung injury improvement effects, and greatly reduced mortality (Degner et al., 2018; Kumar et al., 2018; Kumar N. P. et al., 2019; Li et al., 2021; Wang Y. et al., 2022). In addition, metformin also could induce autophagy, regulate oxidative stress, and enhance the efficacy of anti-TB drugs (Fatima et al., 2021).

Currently, a prospective, randomized, open-label phase II clinical trial is being performed in PTB patients with HIV to explore the safety and tolerability of metformin in combination with standard anti-TB treatment (ClinicalTrials.gov Identifier: NCT04930744).

### 3.7 Loperamide

Loperamide, an inexpensive, over-the-counter, mu-opioid receptor agonist, is commonly used as an antidiarrheal agent. Loperamide could significantly inhibit the growth of *Mtb in vitro* and *in vivo*, and regulate the expression of autophagy-related genes after *Mtb* infection (Juárez et al., 2016; Barrientos et al., 2021). It could also induce overexpression of bactericidal/permeability-increasing protein (BPI) and antimicrobial peptide LL37 genes, increasing the ability of macrophages to eliminate bacterial infection (Juárez et al., 2018). In addition, loperamide induced the production of μ-opioid receptor-dependent antimicrobial peptides, decreased the TNF-α level and increased the production of IL-10 and prostaglandin E2 in macrophages infected with *Mtb* (Wetzel et al., 2000; Happel et al., 2011), and blocked calcium channels to reduce the production of pro-inflammatory cytokines in the body, thus preventing tissue damage caused by increased inflammation (Ye et al., 2012). Therefore, loperamide has a potential immunotherapeutic effect on TB.

### 3.8 Salazosulfapyridine

Salazosulfapyridine (SASP) is now recognized to be a useful agent in the management of inflammatory bowel disease (Crohn’s disease and ulcerative colitis). The 5-aminosalicylic acid produced by SASP in the presence of intestinal microorganisms has antibacterial and anti-inflammatory effects and can inhibit the synthesis of inflammatory mediators such as prostaglandin and leukotriene (Kang et al., 2017). However, SASP could enhance the *Mtb* clearance activity of macrophages by activating NADPH oxidase to promote the oxidation of mycothiol in *Mtb* macrophages, rather than the metabolite-producing 5-aminosalicylic acid (Cai et al., 2016). Previous studies have shown that SASP could significantly reduce the *Mtb* bactericidal load and histopathological inflammation of lungs in mice, and prevent severe inflammation induced by *Mtb* infection of human macrophages (Cai et al., 2016; Wang Y. et al., 2022).

Currently, two interventional studies are being conducted at the Shenzhen Third People’s Hospital (Shenzhen, China), to verify the effect of all-oral short-course regimens containing SASP on extending or shortening treatment duration in MDR-TB patients (Chictr.org.cn: ChiCTR 2,100,045,930 and ChiCTR2000032298).

### 3.9 Glucocorticoid

Glucocorticoids, a kind of primary stress hormone with potent anti-inflammatory and immunosuppressive effects produced by the adrenal cortex, act throughout the body to treat autoimmune diseases, asthma, cancer, and TB. Glucocorticoids could reduce leakage and inflammatory cell infiltration, stabilize lysosomal membrane, protect the mitochondria, relieve bronchospasm, improve pulmonary ventilation, and could also reduce local lesion of tuberculous immune pathological injury and serous effusion, and reduce mortality in the treatment of TB (Schutz et al., 2018). However, further research is required to ascertain the potential immunomodulatory effects of glucocorticoids.

In clinical practice, glucocorticoids are often used in the adjuvant treatment of TBM, tuberculous pleurisy, tuberculous peritonitis, caseous pneumonia, and hematogenous disseminated TB. The administration of Prednisone within the initial 4 weeks following the initiation of Antiretroviral Therapy (ART) for HIV infection demonstrated a reduced occurrence of tuberculosis-associated Immune Reconstitution Inflammatory Syndrome (IRIS) compared to the administration of placebo (ClinicalTrials.gov Identifier: NCT01924286) (Meintjes et al., 2018). Recently, it has been demonstrated that adjunctive dexamethasone has no benefit in improving the survival of HIV-positive adults with TBM (ClinicalTrials.gov Identifier: NCT03092817) (Donovan et al., 2023). Another phase III, randomized, double-blind clinical trial in TBM patients is conducted in Vietnam to determine whether the Leukotriene A4 hydrolase (LTA4H) genotype defined the efficacy of dexamethasone in HIV-uninfected TBM patients (ClinicalTrials.gov Identifier: NCT03100786). Besides, the other randomized control trial is also being performed by National Taiwan University Hospital to investigate the effect of prednisolone as an adjunct therapy to improve the symptoms worsening in patients with tuberculous lymphadenitis (ClinicalTrials.gov Identifier: NCT05861440).

### 3.10 Thalidomide

Thalidomide, a potent agent with teratogenic effects, has proven to be useful and effective in refractory dermatologic disorders. As a glutamic acid derivative, thalidomide has immunomodulatory and anti-inflammatory effects. Thalidomide exerted an immunomodulatory function through the inhibition of TNF-α, elevation of IFN-γ levels, and regulation of NF-κB, IL-6, and IL-1 secretion, along with the modulation of COX-2 and prostaglandin E2 production (Fu and Fu-Liu, 2002; Van Toorn et al., 2021a). Besides, thalidomide synergistically stimulated CD8^+^ T cells more than CD4^+^ T cells (Fu and Fu-Liu, 2002). Thalidomide could be used as an immunosuppressant in the adjuvant treatment of TB patients with severe TB immune response or immunopathological damage, or patients with complex central nervous system TB. A cohort study from South Africa showed that thalidomide was safe, well tolerated, and had a modest clinical effect in patients with central nervous system TB-related complications (Van Toorn et al., 2021b). However, another review showed that thalidomide had no significant clinical improvement in patients with central nervous system TB compared to the control group and had numerous adverse reactions, while it appeared to be effective in HIV-positive patients with TB-immune reconstitution inflammatory syndrome (Panda et al., 2021). In conclusion, it is important to note that thalidomide can cause severe teratogenic effects and should be used with caution in clinical trials. Currently, a randomized, active-controlled phase II and III clinical trial is performed by PGIMER Chandigarh to study the efficacy of thalidomide in complicated tubercular infection of the central nervous system (Ctri.nic.in: CTRI/2023/05/052417).

### 3.11 Other drugs with immunotherapeutic effects

Furthermore, aside from the above drugs, certain pharmaceutical agents employed in the treatment of alternative diseases exhibit immunotherapeutic properties toward TB. For example, anticancer drugs (everolimus, temsirolimus, ridaforolimus) could increase the ability of macrophages to fight *Mtb* by inhibiting the rapamycin (mTOR) pathway and promoting autophagy (Cerni, et al., 2019). A prospective, open-lable, randomized, phase II trial demonstrated that everolimus was safe, and well tolerated as an adjunctive treatment for TB (ClinicalTrials.gov Identifier: NCT02968927) (Wallis et al., 2021). However, a case report demonstrated that the administration of everolimus did not yield any discernible impact on the viability of *Mtb*, and it did not induce an elevation in intracellular reactive oxygen species production within infected macrophages (Bianco et al., 2023). Imatinib, a drug for the treatment of leukemia, could promote autophagy and acidification, synergistic enhancement of the anti-TB effect of RIF, reduce the survival of *Mtb* in macrophages, and have a certain effect on the treatment of MDR and XDR-TB (Napier et al., 2011). Recently, to evaluate the safety, pharmacokinetics, and effects of imatinib on myelopoiesis in TB patients when given with and without INH and rifabutin, a phase II, randomized clinical trial has been completed by National Institute of Allergy and Infectious Diseases (NIAID, Atlanta, Georgia, United States) but with no results published (ClinicalTrials.gov Identifier: NCT03891901). The anti-AIDS drug saquinavir could effectively improve the expression of HLA class II antigen, induce the proliferation of CD4^+^ T cells, promote the secretion of IFN-γ, and significantly kill intracellular *Mtb* (Pires et al., 2021), which is currently in the experimental stage *in vitro*. Zileuton, used for the treatment of asthma and allergic rhinitis, was able to inhibit the production of pro-inflammatory leukotriene and increase the level of IL-1β. It also possessed some degree of tracheal protection, tracheal dilation, and anti-inflammatory effects, and could reduce the burden and pathology of *Mtb*-infected mice, and improve the survival rate (Mayer-Barber et al., 2014; Mishra et al., 2020). However, the research is still at the preclinical stage.

## 4 Conclusion and prospects

### 4.1 Issues and approaches

Enhancing protective immunity and decreasing pathological damage may play an important role in TB treatment. Immunologically active substances, including natural active compounds, small molecule compounds, and repurposed drugs, have achieved satisfactory results *in vitro* experiments, animal experiments, or clinical trials. However, there are still few immunologically active compounds really used in the clinical adjuvant treatment of TB patients, possible reasons for this phenomenon might include the following.

1. Etiopathogenesis of TB is intricate and complex. On the one hand, the host will form granulomas after a large amount of *Mtb* invades the body, the inflammation caused by delayed hypersensitivity will encase and remove the bacteria in macrophages. On the other hand, this excessive inflammation may cause a pathological state of targeted tissues or organs. Therefore, the question of how to effectively use the immune methods for immune regulation and intervention, promote physiological response, and inhibit pathological effects, is a direction for future research.
2. The discovery and preclinical study of immunologically active compounds are relatively slow. The traditional high-throughput screening methods have been far from meeting the needs of drug research and development, and the early-stage research and development strategies of small molecule compounds and natural products need the effective combination of many modern methods. For example, recent years have seen great advances in the development of new drug screening methods such as proteomics, genomics, quantum computing, CRISPR, and artificial intelligence. The application of multiple screening methods may provide more possibilities for discovering more immunologically active compounds (Ejalonibu et al., 2021).

Admittedly, several questions need to be discussed critically about the immunomodulatory candidates. Firstly, the lack of pharmacokinetic data for immunologically active compounds and the corresponding modified compounds in the TB population is a limitation, including absorption, distribution, metabolism and excretion parameters (for example, the bioavailability, plasma protein binding rate, half-life, etc. of the compound in serum. And some available data on the site of infection). Pharmacokinetic data would have assisted in interpreting potential drug–drug interactions for the regimen. Secondly, studies on the possible drug–drug interactions between immunologically active compounds and current first-line anti-TB drugs have not been through enough, and the drug interaction mechanisms are not clear. Finally, oral administration is the best option for TB patients who need long-term treatment. However, at present, most oral preparations of immunologically active compounds have not been used in animal experiments or clinical trials, which will be a new challenge in the future. Besides, studies on the effect of bioavailability differences caused by different routes of administration on adjuvant therapy are not comprehensive. Therefore, for lead compounds that are promising as candidate drugs, preclinical studies should be accelerated to obtain more pharmacodynamics and toxicological data through animal experiments, paving the way for subsequent clinical trials.

1. The clinical application of immunomodulators is not standardized. To date, there remains no consensus as to the selection, dosage, application time, treatment courses, and the intervention effect of immunomodulator. Besides, appropriate immunomodulator selection strategies and safe and effective combination therapy regimens have not been developed. For instance, should immunomodulatory drugs deemed appropriate for incorporation into conventional anti-TB therapy be administered concurrently with standard treatment, or should the therapeutic approach be customized to suit the individual patient? Furthermore, what are the enduring detrimental impacts of these repurposed drugs on the host subsequent to their utilization in immunomodulatory therapy? Additionally, what is the risk of resistance emergence? Further studies are required to explore these issues. Therefore, there is a significant need to speed up the pace of drug clinical study and the patient-specific application of these immunomodulators in the clinic.

### 4.2 Future perspective

The WHO emphasizes the importance of reducing the treatment duration for both sensitive and drug-resistant TB. The use of immunomodulators is deemed necessary, with glucocorticoids in combination with anti-TB medications showing significant improvements in patients suffering from tuberculous meningitis, tuberculous pericarditis, tuberculous pleurisy, and other related conditions (Jianqin, 2022). However, it is crucial to carefully select appropriate immunomodulators to effectively stimulate the host immune system and produce protective effects, considering the dual nature of the immune response triggered by *Mtb*. Hence, the future of immunoadjuvant therapy for TB may center on the advancement of host-directed therapy and its corresponding pharmaceutical agents (Chinese Society of Tuberculosis CMA, 2022) to achieve personalized, diversified and multifunctional immunotherapy. For instance, enhancing research on novel immune activation and suppression products, investigating immunotherapy based on intestinal flora, and exploring new approaches to immune agents mediated by nanotechnology can contribute to advancements in the field. This necessitates interdisciplinary collaboration across fields such as molecular biology, immunology, pharmacy, and clinical medicine to expedite clinical research, amass evidence-based data for the clinical utilization of immunomodulators, and identify novel targets for host immune-assisted therapy. These efforts aim to establish a more robust scientific foundation for the future development of immunomodulatory drugs.

In conclusion, while the efficacy of MDR/XDR-TB treatment is often constrained, various immunomodulators showed promise in preventing latent *Mtb* reactivation, avoiding active *Mtb* spread, mitigating adverse reactions to anti-TB drugs, shorten chemotherapy duration, and improving cure rates. Nevertheless, the intricate interplay between host and pathogen may also heighten the likelihood of disease progression or exacerbation of inflammation. Therefore, prospective multi-center large-scale and multidisciplinary in-depth investigations are imperative to yield substantial practical value in the utilization of immunoregulatory agents for TB treatment.

## Author contributions

JM: Investigation, Writing – original draft. XW: Supervision, Writing – review & editing. JL: Supervision, Writing – review & editing.
